# *Pasteurella dagmatis* prosthetic valve endocarditis with suspected mycotic aortic aneurysm: a case report

**DOI:** 10.1093/omcr/omag126

**Published:** 2026-07-12

**Authors:** Qais Akasheh, Tania Lasrado, Paul Carter, John Li

**Affiliations:** Cardiology Department, Ipswich Hospital, Heath Road, Ipswich, Suffolk, IP4 5PD, United Kingdom; Cardiology Department, Ipswich Hospital, Heath Road, Ipswich, Suffolk, IP4 5PD, United Kingdom; Section of CardioRespiratory Medicine, The Victor Phillip Dahdaleh Heart and Lung Research Institute, Cambridge Biomedical Campus, Papworth Road, Cambridge CB2 0BB, United Kingdom; Cardiology Department, Ipswich Hospital, Heath Road, Ipswich, Suffolk, IP4 5PD, United Kingdom

**Keywords:** infective endocarditis, *Pasteurella dagmatis,* prosthetic valve, aortic root abscess, ascending aortic aneurysm, pacemaker

## Abstract

We report a case of prosthetic valve infective endocarditis caused by *Pasteurella dagmatis* in a 79-year-old man with a mechanical aortic valve and a known ascending aortic aneurysm. He presented with fever and malaise, and blood cultures grew *P. dagmatis*. Transoesophageal echocardiography demonstrated a prosthetic valve vegetation with a suspected aortic root abscess, while computed tomography confirmed a large ascending aortic aneurysm. The clinical course was complicated by conduction disturbances requiring permanent pacemaker implantation. The patient underwent urgent surgical intervention and made a good recovery, being discharged in a stable condition, but with persistent atrial fibrillation. This case highlights the rarity of *P. dagmatis* as a cause of IE and its potential for invasive complications. Although histopathological confirmation was not obtained, the infection may have contributed to progression of the pre-existing aneurysm, raising the possibility of a superimposed mycotic aneurysm.

## Introduction

Infective endocarditis is a microbial infection of the endocardial surface of the heart, most commonly involving native or prosthetic heart valves. It may also affect intracardiac devices or areas of endocardial damage. A hallmark of the disease is the formation of vegetations, which are masses of platelets, fibrin, and inflammatory cells formed in response to microbial invasion. Early detection and initiation of appropriate therapy are crucial to minimize the risk of severe outcomes, including cardiac failure, embolic events, and death. Its epidemiology has evolved over recent decades, shifting from a disease predominantly affecting younger patients with rheumatic heart disease to one more commonly seen in older individuals with multiple comorbidities, prosthetic valves, and healthcare-associated risk factors [1]. Zoonotic organisms are rare causes of IE, and *Pasteurella* species, which are typically associated with animal bites or soft tissue infections, are an uncommon aetiology, accounting for approximately 10.7% of cases. Human infections by *Pasteurella* spp. are also known to occur in patients without a history of animal bite but with close animal contact [2]. To our knowledge, this is the first reported case of *P. dagmatis* causing IE with a mycotic aortic aneurysm, highlighting its potential for severe invasive complications.

## Case presentation

We present the case of a 79-year old male, of Greek origin, who was admitted to hospital with a 2-day history of fever (39.6°C) and fatigue. His past medical history included transient ischemic attack, chronic inflammatory demyelinating polyneuropathy, hypertension, and previous mechanical aortic valve replacement for severe bicuspid aortic stenosis.

On admission, inflammatory markers were mildly elevated and chest radiography was unremarkable. *P. dagmatis* was identified in both aerobic and anaerobic bottles from one blood culture set using MALDI-TOF mass spectrometry. Following microbiology laboratory discussion, contamination was considered unlikely, particularly as growth occurred in both bottles. He owned a pet dog but denied history of bites or scratches.

Transthoracic echocardiogram demonstrated preserved left ventricular systolic function with an ejection fraction of 55%, a well-seated prosthetic aortic valve, multiple echogenic reflections on the valve, dilation of the aortic sinus (4.5 cm), and enlargement of the proximal ascending aorta (5.6-5.9 cm). IE could not be excluded, and an urgent transoesophageal echocardiogram (TOE) was performed.

TOE demonstrated a 9 mm vegetation on a mechanical aortic valve prosthesis, with an echolucent space surrounding the aortic valve and root (Fig. 1) and thickening of the ascending aorta. The proximal ascending aorta was dilated to 52 mm. These appearances were highly suggestive of an aortic root abscess and an ascending aortic aneurysm.

**Figure 1:**
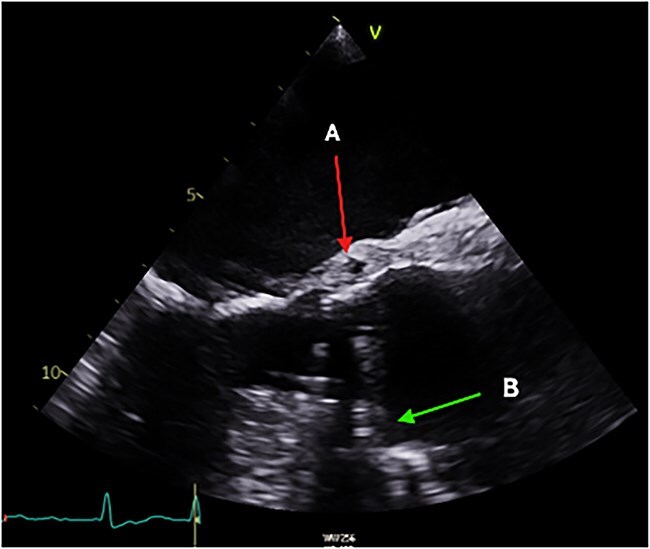
Transoesophageal echocardiography imaging of the aortic valve. Arrow A: Aortic root abscess. Arrow B: Vegetation.

The diagnosis of definite IE was confirmed using the Modified Duke’s criteria, fulfilling one major criterion (positive echocardiogram) and three minor criteria (fever > 38°C, predisposing condition, microbiological evidence).

Continuous cardiac monitoring during admission revealed slow atrial fibrillation with long pauses, requiring permanent pacemaker implantation. These conduction abnormalities were deemed secondary to IE complicated by an aortic root abscess.

Computed tomography (CT) imaging confirmed a 7 cm ascending aortic aneurysm without evidence of dissection (Fig. 2). The discrepancy in aneurysm size between TOE and CT likely reflects the imaging limitations of TOE, which primarily visualises the proximal ascending aorta, whereas CT provides more comprehensive evaluation of the entire ascending aorta and can detect more distal dilatation.

**Figure 2:**
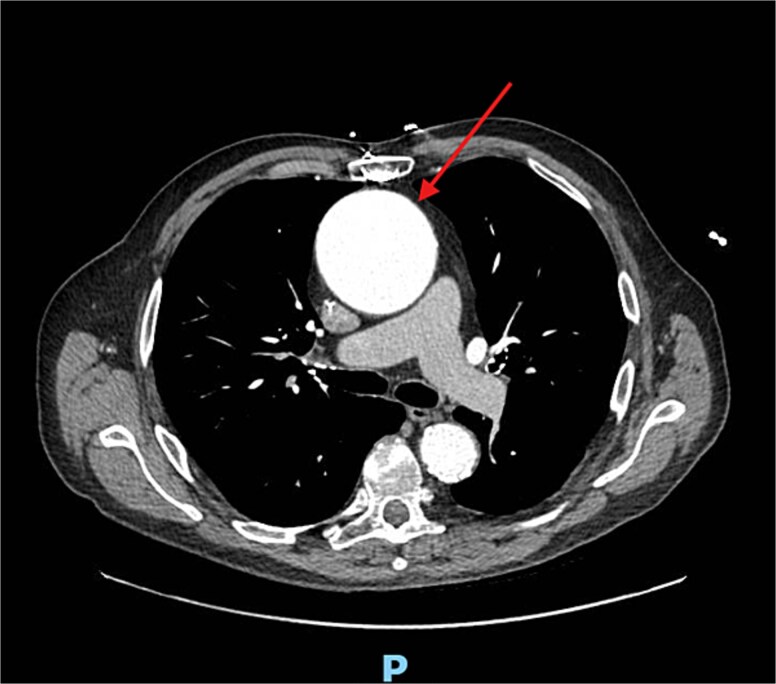
Contrast-enhanced computed tomography of the aorta demonstrating an ascending aortic aneurysm measuring 7 cm in diameter.

In light of these findings and the significant aneurysm size, the patient was referred to the nearest cardiothoracic surgical centre, where he underwent emergency surgery. Intraoperative findings included a dilated ascending aorta of poor tissue quality with significant calcification distally and of the aortic root involving shelves around the aortic annulus and above the coronary ostia. A sub-annular abscess extended through the anterior mitral valve leaflet below the non-coronary cusp. The aortic valve was carefully resected and left ventricle cavity and aortic root washed out removing all sutures and valve debris. The ascending aorta was replaced with a 34 mm woven Dracon graft and a new bipolar right ventricle pacing system was placed to mitigate the risk of infection involving the existing system and potential need for extraction. The patient was discharged on postoperative day 10 with a further 4-week course of amoxicillin. At an outpatient follow-up 2 months later, he remained clinically well and asymptomatic. Review of the pacemaker demonstrated 17% ventricular pacing; however, he was not pacemaker dependent.

## Discussion

We report a rare case of *P. dagmatis* prosthetic valve IE involving a mechanical aortic valve, complicated by aortic root abscess, conduction system involvement requiring permanent pacing, and a large ascending aortic aneurysm suspicious for mycotic aneurysm formation. This case expands the limited literature on *P. dagmatis* infection, highlighting its potential for severe, invasive complications; which is important given the high morbidity and mortality of IE, particularly in patients with prosthetic valves [1].

*Pasteurella* species are Gram-negative organisms typically associated with animal exposure. Similarly to this case, overt bites or scratches are not always reported [2, 3], supporting the need for careful exposure history even when there is no obvious inoculation event. Most published cases of *Pasteurella*-related IE involve *Pasteurella multocida*, while *P. dagmatis* remains exceptionally rare [4–7]. This case, which included microbiological confirmation, adds to the limited literature supporting *P. dagmatis* as a true causative pathogen in prosthetic valve endocarditis.

To our knowledge, this is the first reported case linking *P. dagmatis* IE with multiple severe complications, including suspected mycotic ascending aortic aneurysm formation. This is supported by marked aortic dilatation, together with extensive infection demonstrated intraoperatively and on multimodal imaging. However, histopathological confirmation would have been required for a definitive diagnosis. Otherwise, the peri-annular abscess, along with new conduction abnormalities, are recognised markers of invasive disease and poor prognosis in prosthetic valve endocarditis. The observed bradyarrhythmia likely reflected extension of infection into the conduction system, underscoring the need for urgent cardiothoracic referral. Multimodal imaging was critical: TOE enabled detailed assessment of prosthetic valve involvement, while CT delineated the extent of aortic pathology.

This rare association broadens the recognised clinical spectrum of *P. dagmatis* infection and highlights its potential to cause destructive endovascular complications requiring urgent combined medical and surgical management.

## References

[1] Mills MT, Al-Mohammad A, Warriner DR. Changes and advances in the field of infective endocarditis. Br J Hosp Med 2022;83:1–11. 10.12968/hmed.2021.0510 35377207

[2] Alifragki A, Kontogianni A, Protopapa I. et al. Infective endocarditis by Pasteurella species: a systematic review. J Clin Med 2022;11:5037. 10.3390/jcm11175037 36078964 PMC9456824

[3] Porter RS, Hay CM. Pasteurella endocarditis: a case report and statistical analysis of the literature. Case Rep Infect Dis 2020;2020:8890211. 10.1155/2020/8890211 32765917 PMC7387957

[4] Al GMA, Abba AA, Al NM. Endocarditis caused by Pasteurella multocida. Ann Saudi Med 2006;26:147–9. 10.5144/0256-4947.2006.147 16761455 PMC6074155

[5] Rottmann FA, Schorle P, Giesen R. et al. Prosthetic valve endocarditis caused by Pasteurella dagmatis, Germany. Emerg Infect Dis 2024;30:2202–4. 10.3201/eid3010.240727 39320237 PMC11431900

[6] Rosenbach KA, Poblete J, Larkin I. Prosthetic valve endocarditis caused by Pasteurella dagmatis. South Med J 2001;94:1033–5. 10.1097/00007611-200194100-00020 11702818

[7] Sorbello AF, O'Donnell J, Kaiser-Smith J. et al. Infective endocarditis due to Pasteurella dagmatis: case report and review. Clin Infect Dis 1994;18:336–8. 10.1093/clinids/18.3.336 8011812

